# Live Spoofing Detection for Automatic Human Activity Recognition Applications

**DOI:** 10.3390/s21217339

**Published:** 2021-11-04

**Authors:** Viktor Dénes Huszár, Vamsi Kiran Adhikarla

**Affiliations:** 1 Teqball Kft., Expo tér 5-7, 1101 Budapest, Hungary; 2 Doctoral School of Military Engineering, National University of Public Service, Ludovika tér 2, 1083 Budapest, Hungary

**Keywords:** deep learning, human activity recognition, spoof detection, spoof attack database, security, smart cities

## Abstract

Human Activity Recognition (HAR) has become increasingly crucial in several applications, ranging from motion-driven virtual games to automated video surveillance systems. In these applications, sensors such as smart phone cameras, web cameras or CCTV cameras are used for detecting and tracking physical activities of users. Inevitably, spoof detection in HAR is essential to prevent anomalies and false alarms. To this end, we propose a deep learning based approach that can be used to detect spoofing in various fields such as border control, institutional security and public safety by surveillance cameras. Specifically, in this work, we address the problem of detecting spoofing occurring from video replay attacks, which is more common in such applications. We present a new database containing several videos of users juggling a football, captured under different lighting conditions and using different display and capture devices. We train our models using this database and the proposed system is capable of running in parallel with the HAR algorithms in real-time. Our experimental results show that our approach precisely detects video replay spoofing attacks and generalizes well, even to other applications such as spoof detection in face biometric authentication. Results show that our approach is effective even under resizing and compression artifacts that are common in HAR applications using remote server connections.

## 1. Introduction

With the availability of ubiquitous computers and smart wearable sensors, Human Activity Recognition (HAR) has become one of the popular research topics in recent years. HAR algorithms aim to present information on simple or complex physical activities of humans. More commonly, these algorithms take data from various sensors as input and use machine learning and computer vision techniques to extract information on human activities. Consequently, HAR can be extensively used in several applications such as medical diagnosis, keeping track of elderly people [1,2,3], smart homes [4], automated driving [5], military training and surveillance to control crime rates [6,7] and motion-driven virtual games [8]. In the context of automatic HAR, spoofing attack represents a situation when a person misleads the activity recognition algorithm by providing disguised or copied visual data and the algorithm reports these fake data as a successfully performed action. Currently, there is a great demand for intelligent video surveillance in places such as, but not limited to, large waiting rooms and campuses to recognize simple or complex motion patterns and derive high-level subjective descriptions of actions and interactions among subjects and/or objects. Nowadays, most people are equipped with smart phones and spoofing attacks such as playing a previously recorded video on a smart phone screen in front of a surveillance camera would mislead or divert such intelligent systems. Therefore, there is a need for detection of spoofing attacks of this kind before making a final decision on the recognized action.

**Figure 1:**
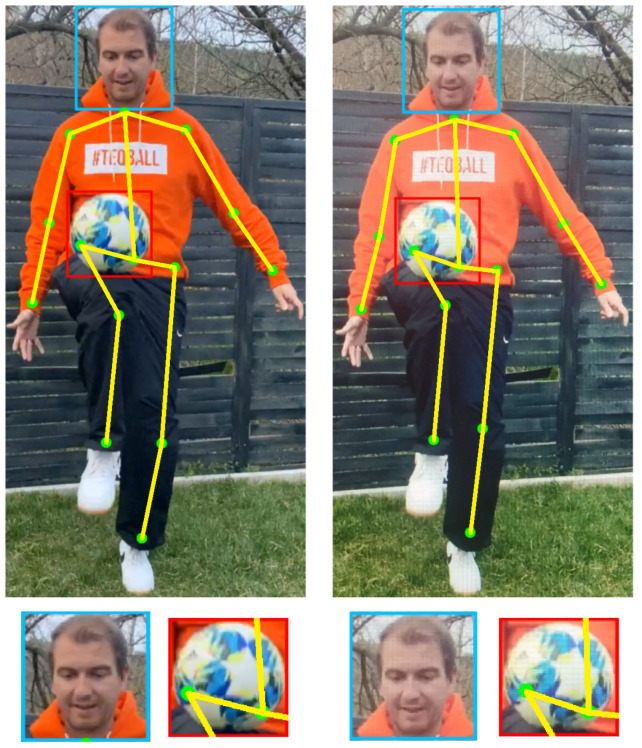
A sample event detection in a digital football game—the user hits the ball with the right knee. Top row: left—detection on a single frame of a video that captured a real user, right—detection on the same frame of the same video captured on a computer monitor (fake user). Bottom row left to right: close-ups of the detected head and football of real and fake users respectively.

Sensors used for HAR can be broadly classified into external and wearable sensors [9]. Wearable sensors measure data required for activity recognition while being in physical contact with the user. Examples for wearable sensors are accelerometers, gyroscopes, and magnetometers which are used to translate human motion into signal patterns for activity recognition [10]. In the case of external sensors, the sensors are set at fixed points, and users are expected to interact with them (common in applications such as public safety by surveillance cameras). With the evolution of deep learning methods such as convolutional neural networks and recurrent neural networks, it is possible to achieve state-of-the-art results by automatically learning features from the raw sensor data. A use-case that employs deep learning approaches for HAR using external sensors is shown in Figure 1. Here, a user, with the help of a football, is interacting with a digital gaming application that aims to analyze and rate the freestyle football/soccer performance of the user. Specifically, deep learning algorithms are employed for detecting body parts together with a football in real-time. The real-time detection information enables us to recognize the current activity of a user for our use-case. Subsequently, the result of this detection enters the evaluation stage, which lets the user compare their skills to others and try to improve their abilities.

Despite the high recognition accuracy, HAR systems are not capable of distinguishing between real and fake humans, e.g., human videos played on a mobile screen or on a computer monitor. Intruders can utilize a wide variety of mediums to launch spoof attacks. One of the most common ones is replaying videos on digital screens. Figure 1 demonstrates this by showing the detection performed by the same deep learning algorithm on a real user video and a replayed video. Thus, HAR systems are susceptible to spoof attacks that can cheat these systems to recognize a fake user as a genuine user. One of the most important security challenges is that the fraud and spoof detection must be designed in accordance with existing infrastructural systems. Anyone planning a fraudulent, criminal activity may have innovative ideas and solutions concerning how to circumvent machine/computer vision, in order to bypass the simplest security gates and possibly break through computer vision watchdogs. Therefore, it is very important for future developments to effectively filter out such potential frauds.

The challenge is that the capabilities of existing cameras may not be able to always provide good quality data due to weather and visibility conditions. It is a challenge that it is naturally difficult as it involves large amounts of data for teaching CNN-based algorithms and due to the General Data Protection Regulation (GDPR) and other regulations, data sources can be limited. In particular, in cases like military training and surveillance, data are often classified and access to them is limited by nature.

Therefore, by using artificial intelligence, profiling competencies based on pre-defined risk criteria can be developed by processing and analyzing image information from existing installed cameras. The basis of image analysis is object analysis, motion analysis etc. Furthermore, the verification of the data could be carried out with the support of the competent authorities e.g.,: the police or National Security Special Service. Defining the professional requirements and methodology of further experimental development and processing the results of the feasibility phases can be the task of national public service universities, military and police academies.

To safely use automated HAR systems, spoof detection techniques are required to detect spoof attacks before performing recognition. In the context of HAR, users are always moving and, depending on the speed of the motion, this can generate blurred images on captured video which makes the spoof detection challenging in such applications. Moreover, depending on the complexity of the action recognition, it is sometimes needed to stream visual data to a server for remote processing. This might involve video compression and video resizing operations that degrade the quality of the video and makes the task of spoof detection even harder. To address these issues, in this work, we propose a deep learning-based approach for spoof detection on videos for HAR applications in such challenging conditions.

**Figure 2:**
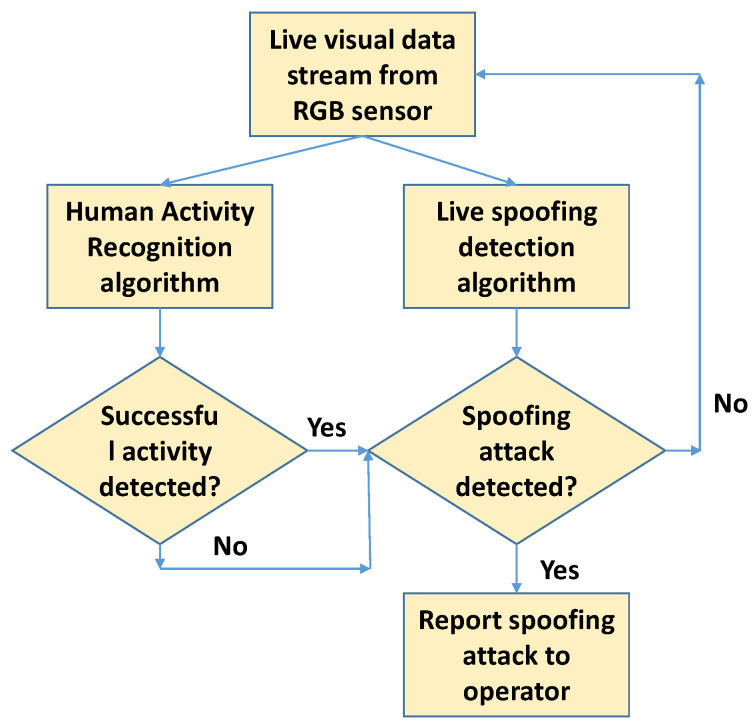
Proposed workflow for spoofing detection in HAR applications: a lightweight spoofing detection algorithm is run in parallel to the HAR algorithm for live detection of spoofing attack cases.

In the current work, we propose a lightweight deep learning-based algorithm that runs in parallel with HAR algorithms to detect and report cases of spoofing. Figure 2 shows the flowchart of our proposed system architecture. Incoming real-time visual data stream are fed in parallel to HAR and proposed spoofing detection algorithms. Irrespective of a successful or unsuccessful activity recognition using the HAR algorithm, on positive detection of a spoof case, the spoofing attack is reported to the operator. If no spoofing attack is detected, further visual data are grabbed from the sensor for processing by HAR and spoofing detection algorithms. In particular, the contributions of this work are:

1. A deep learning-based approach that can be used to detect spoof attacks from video frames capturing humans. The algorithm precisely detects the spoofing attacks and is also able to cope with video resizing and streaming artifacts, while it remains lightweight enough to run it on a mobile device alongside the main HAR algorithms.
2. A strategy to combine detection of the proposed deep learning network, temporally on a captured video or on a live video stream, for detection of video replay spoof attacks systematically while maintaining real-time performance.
3. A new database comprising real and spoof videos captured from 30 different users in different locations and under different lighting conditions. The database is diverse and precisely captures the required features for training our deep learning network.
4. An additional evaluation of the performance of the proposed approach in the context of bio-metric recognition applications.
5. An IOS mobile application that implements the proposed approach in real-time on a mobile device.

The rest of the paper is organized as follows: In Section 2, we discuss the related works. Section 3 describes the proposed approach in detail. In Section 4, we present the results and discuss the scope of applicability of our approach to other application domains. In Section 5, we conclude the work and derive future directions for the proposed approach.

## 2. Related Work

There is a vast variety of techniques available for detecting replay attacks from visual data. Most of these address spoofing detection in bio-metric recognition applications, predominantly using facial image analysis. Users are located close to the camera and looking towards the camera. To the best of our knowledge, our system is the first of its kind exploring spoof detection from videos in the context of HAR where users are located far away from the camera and are not always looking at the camera. In the subsequent paragraphs, we present some of the more relevant works in the domain of spoof detection using facial image data.

In the field of replay attack detection, techniques proposed in the literature can be grouped into four major groups: user behavior modeling, user cooperation, hardware-based and data-driven characterization [11].

Behavior modeling techniques aim to trace user actions such as movement of the head and blinking. They are used to understand if a person in front of the camera is a real user or just a photograph of them [12,13]. Other branches of such methods detect subtle movements of a live human face using motion magnification [12]. These are not relevant to our current work since they are only effective if the attacker uses a photograph. In contrast, our main target is to detect video replay attacks.

User cooperation-based techniques look for presentation attacks by exclusively initiating a secondary interaction between the user and the detection module [14]. One such example is prompting the user to perform a certain movement or action. In applications related to automated video surveillance, it is not practical to use these user cooperation-based techniques since the users may not deliberately interact with the system.

Hardware-based techniques make use of additional hardware such as infrared or depth sensors to understand the depth information of the scene [15,16]. Within such techniques depth cues enable us to differentiate between a flat object such as a mobile telephone screen or real 3D objects such as humans. Thus, these methods provide better robustness against variations in illumination and pose of the user. However, in applications such as motion-driven games designed to work with smartphones, users may not always possess such additional hardware on their devices. Thus, the scope of the application for these methods is limited.

Finally, there are techniques that are data-driven and characterize the replay attacks by predicting/learning the artifacts/features of attempted attacks using the visual data that came from a standard acquisition sensor. This work focuses on such data-driven techniques using visual data obtained from fixed or mobile cameras. In the rest of this section, several techniques that are data-driven and relevant to the current work are discussed.

### 2.1. Depth Analysis

There are a branch of techniques for spoofing detection based on scene depth analysis using optical-flow estimation. They intend to estimate the 3D structure of the face of a user to discriminate between a 3D live face and a 2D spoof face [17,18]. Similar to depth camera-based spoofing detection, live faces are 3D objects, and can be clearly differentiated from a 2D planar medium such as photographs. Such methods can be practically used to identify attacks from planar static mediums. However, if the camera is not stationary or if the user is moving, obtaining reliable depth information for performing spoof detection can be very difficult and challenging. Moreover, depth or shape analysis examines several frames to make a single prediction which can make these approaches slow and resource-intensive.

### 2.2. Texture Analysis

The mediums used for spoofing such as paper or digital screens have different reflection properties than real and live faces. Texture analysis approaches make use of this observation for spoofing detection [19,20,21]. By modeling the interaction between the illuminating and the reflective surfaces, it is possible to extract albedo and normal maps [22], which can be used as features to discriminate between real and attack samples. These methods have faster response time than depth analysis-based approaches since they often examine one frame at a time. However, they have poor ability to generalize to HAR applications, since there can also be reflective objects in real videos which can trick such approaches. Furthermore, in the case of HAR applications, users are dispersed and the lighting is mixed and uncontrollable. So, the basic assumptions do not hold in practice. Other texture-based approaches try to capture the high frequency information using descriptors, such as a variation of local binary patterns (LBP) [23,24].

### 2.3. Image Quality

These methods estimate degradation in overall image quality which occurs during recapturing of photographs or screens. Some factors that contribute to image quality degradation include blurriness and color deformation [25,26,27,28]. To compute image quality, it is often necessary to have a reference image which is not available. The usual approach is to simulate a degraded version of the source image and use it together with the source image for computing image quality. Here the hypothesis is that the objective quality degradation between the source and the simulated image is relatively less in the cases of real images when compared to attack images. Thus, if acquisition conditions are not similar, this hypothesis does not hold, which makes these methods delicate in HAR scenarios.

### 2.4. Frequency Domain Analysis

Recapturing on digital spoofing mediums introduces high frequency noise into the image data due to the discreteness of the presentation mediums. This frequency specific information can be captured by Fourier analysis [29,30]. Frequency domain-based methods explore these noise signals in recaptured video to distinguish between live and spoof faces [31,32]. These noise signals in the frequency domain are strong cues to detect attacks. However, it should be noted that using high-definition spoofing mediums can dampen this noise and the recognizable noise patterns are not always present, which makes such approaches solely based on them unreliable.

### 2.5. Methods Based on Deep Learning

In recent times, in the context of several image recognition applications, it is proven that models based on Convolutional Neural Networks (CNNs) achieved state-of-the-art performances. Such deep learning approaches learn to predict features or intermediate representations directly from the raw pixel data without the need for computing the features explicitly. Using CNNs for performing spoof detection in HAR applications is not represented in the literature. However, architectures are proposed for performing spoof detection especially on videos in facial bio-metric authentication applications. Rodrigo B. et al. [11] proposed an approach using ResNet50 [33]. They train this network using several pre-computed maps such as depth, saliency and illumination maps, which makes their approach context dependent. Using such approaches on a mobile device can be cumbersome, since computing these maps can be highly resource intensive.

Yaojie L. et. al. [34] proposed an approach using a combination of CNNs and Recurrent Neural Networks (RNNs) for detecting spoofed faces. Particularly, they use RGB+HSV representation of images and use several video frames to make a single prediction. We argue that residual networks are usually slower since they work on multiple frames.

Atoum. Y. et. al. [35] introduced a two-stream CNN which computes (a) local features from patches and (b) depth maps and combines the output of these streams for replay attack detection. Using local features makes these methods robust. However, due to depth map computation, they may not be able to keep up with the real-time requirements, especially, when an activity recognition algorithm also has to run in parallel on a mobile device. In the current work, we also target the mobile device scenario where lightweight models are highly favored.

### 2.6. Summary of State-of-the-Art Methods

Table 1 summarizes the performance of methods published in the literature for face spoofing detection on public datasets. Except for deep learning based methods, intra-database results for the Idiap Replay-Attack and UVAD databases are reported using the Half Total Error Rate (HTER) (%) metric. Intra-database results for the CASIA FASD, MSU MFSD and MSU USSA databases are reported using the Equal Error Rate (EER) (%) metric. For the ZJU Eyeblink database, classification accuracy is reported. Unless otherwise specified, all cross-database results are reported using the HTER metric.

Preliminary methods for spoofing detection using approaches such as blinking detection fail under video replay attacks. Other slightly advanced methods based on low-level texture descriptors and simple classifiers for spoofing detection are also shown to fail under challenging cross-dataset protocols [36]. It is important to note that, among the previously developed methods, and most of the modern deep learning methods for video replay spoofing detection, most use public datasets for experimenting with and comparing different methods. We argue that there are several inconsistencies among the existing public datasets for video replay spoofing detection using facial image data in terms of the subject position, image resolution and cameras used for dataset generation. Cross dataset evaluations in the literature have shown limitations of both the developed methods and existing datasets.

We also argue that spoofing detection datasets for biometric or live face detection are not relevant to HAR applications, since they generate data in static sessions with minimum illumination variability. In contrast, in HAR applications, users have the flexibility to freely choose their location and eventually it is not always possible to control the user’s behavior. Such uncontrolled behaviour can happen in school campuses, other institutions and military sites. Moreover, in some cases such as virtual games, some of the body parts including the face are partially or completely occluded by objects for interaction such as a football. Methods developed in the literature for spoofing detection in bio-metric face authentication applications may not extend to such cases. On top of that, if the activity recognition involves remote server processing, the image data may be subjected to resizing and/or compression. The dependency of such operations on spoofing detection accuracy is not discussed in the literature. Finally, the state-of-the art deep learning-based methods for spoofing detection using facial image data involve pre-processing steps such as face alignment after face detection which is impossible in HAR use cases since the head position of a user is not fixed. In addition, in the literature, there are no such meticulous studies using deep learning methods targeted for mobile devices.

To address all of these issues, in this work, we developed new tools including a novel database and robust data-driven approach that jointly examine various regions of the captured images to detect spoofing. Our system enhances and integrates state-of-the-art deep learning algorithms for detecting the replay attack spoof cases for HAR applications.

## 3. Visual Analysis for Video Replay Spoofing Detection

Here, we elaborate our method based on training a CNN model that distinguishes between genuine users and spoof attacks. For experimenting as well as for describing our system, we consider the instance of motion-driven smart phone-based virtual games, in particular, SQILLER [8]. However, we highlight again that our approach is not limited to this specific setting and can be used in higher security-demanding applications such as military grounds, defense and border control. Furthermore, for the sake of simplicity, we consider a single user scenario. Our approach is scalable and can be easily extended to multi-user scenarios.

### 3.1. Context Selection for Spoofing Detection

**Figure 3:**
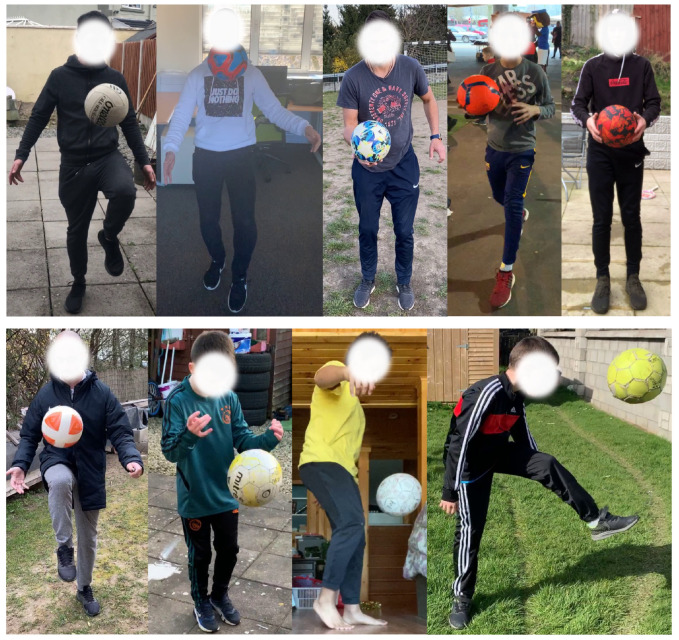
Representative images from our video database. Videos consists of several users playing a digital football game in diverse locations with varying lighting and backgrounds.

Generally, depending on the user activity to be recognized and the complexity of it, activity recognition algorithms involve computing locations of one or several body parts/joints or. in some cases, complete skeleton tracking of a user from an image [45]. In the case of virtual games using an interaction object such as a football, it is also necessary to compute the location of the football in an image together with the user body parts, since here the activity involves interaction of the user with the football (see Figure 1). For this, we created a diverse database consisting of almost 50,500 full HD images of 38 users juggling a football in different backgrounds and lighting conditions. For collecting data, we selected both male and female users between the ages of 8 and 40. These images are extracted from several videos shot using various iPhones—iPhone 6, 6S, SE, 7, 8, X and XS. Some representative images from our database are shown in Figure 3. These images are manually labelled with bounding boxes that encapsulate the following data: human body parts—head, left shoulder, right shoulder, left elbow, right elbow, left hand, right hand, left hip, right hip, left knee, right knee, left foot, right foot and also the bounding box of the football. After gathering the database and labels, we trained the YOLO [46] deep learning CNN architecture and later used it for detecting the required body parts together with the ball from the video frames in a single shot. We chose the well-proven YOLO state-of-the-art CNN for this task because of its fast and robust performance even on mobile devices. Note that, in contrast to traditional face detection methods, we generate face bounding boxes that also contain a substantial background image to provide more contextual information.

In order not to overload the final application for activity recognition with additional functionality of spoofing detection, we consider the already computed body parts location data for this purpose. Among the detected bounding boxes by our trained YOLO model, the head bounding box, comprising of a human face is uncovered most of the time and is composed of different interesting structures and characteristics. Due to the wide usage in bio-metric authentication systems and given its potential in many applications such as surveillance, home and institutional security and border control and military, to experiment further with spoofing detection, we chose to use the extracted head bounding box data from the trained YOLO model. Note that this also ensures generalization of our approach to other HAR applications where there are not any or similar interaction objects and also to cases where the lower human body is not visible.

### 3.2. Models

**Figure 4:**
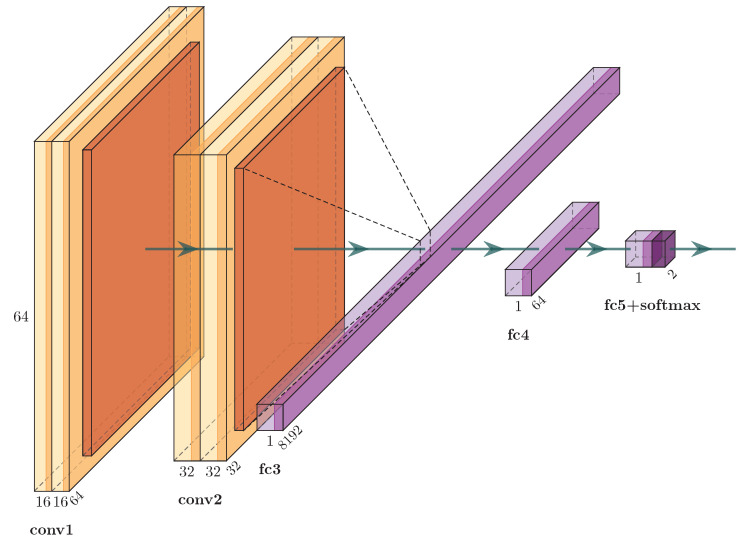
CNN architecture inspired from the VGG16 net used for our experiments.

While working with videos, there are several ways to combine the temporal information which makes it hard to use fixed size architecture. In our work, we handle videos as several short clips consisting of identically sized frames. The idea is to use these several frames of a clip to learn spatio–temporal features. For the experiment, we consider a network inspired by one of the ImageNet challenge-winning models, VGG16 net [47]. We chose this architecture in our experiments for various reasons: firstly, the VGG blocks are smaller networks, supporting real-time performance and are light enough to be embedded into mobile devices. Secondly, it is proven in the literature that VGG16net has the potential to represent complex visual relationships [48]. Finally, the VGG network offers flexibility to change input size and alignment which makes it easier to adopt and modify this network for experimenting with several input data dimensions. Particularly, we consider two VGG blocks, each comprising of two 3 × 3 convolution layers followed by a 2 × 2 pooling layer for experiment. The outputs of these VGG blocks are connected to two fully connected layers. The final layer is connected to a softmax classifier with dense connections (see Figure 4).

**Figure 5:**
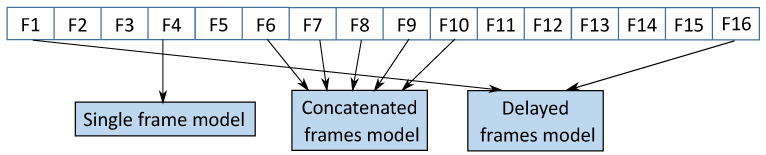
Some of the explored approaches for capturing spatio–temporal features: a single frame model which processes one image at a time; a concatenated frames model which processes consecutive 5 frames at a time; a delayed frames model which processes two frames at a time that are 15 frames apart temporally.

We carefully chose this simple model to achieve better run-time performance while still retaining the detection accuracy. We implemented our system in Keras [49]. We consider the CNN in Figure 4 as baseline CNN and investigate approaches to combine information across temporal domain (see Figure 5). Here we describe our trials learning the spatio–temporal features.

#### 3.2.1. Single Frame Model (SF)

For studying the potential of static frames in accurately classifying the genuine and spoof cases, we consider this model. SF takes every frame of a video and outputs the observations. Here, the extracted face bounding box from the current input Full HD frame is resized to 64 × 64 × 3 pixels and then fed to the aforementioned baseline CNN.

#### 3.2.2. Concatenated Frames Model (CF)

The approach here is to combine the visual information across a time window containing contiguous frames in a video clip. This is achieved by adapting the filters on convolutional layer of the first VGG block in the baseline model. Similar to the SF, the consecutive frames across the considered time window are resized to 64 × 64 × 3 pixels and the initial convolutional filters are extended to be of size 64 × 64 × 3 × N pixels, where N is the size of the considered time window. We choose N = 5 in our work to for learn and detect local patterns across the time window.

#### 3.2.3. Delayed Frames Model (DF)

The delayed frames model uses two separate baseline models until the second VGG block and these two models are fed with two 64 × 64 × 3 resized frames that are P frames apart in the given video sequence. After the second VGG block, the outputs of these two models are merged together and connected to the rest of the fully connected layers on the baseline model. In this work, we use P = 15 for learning and detecting global features.

#### 3.2.4. Ensemble Multi-Stream Model (EM)

**Figure 6:**
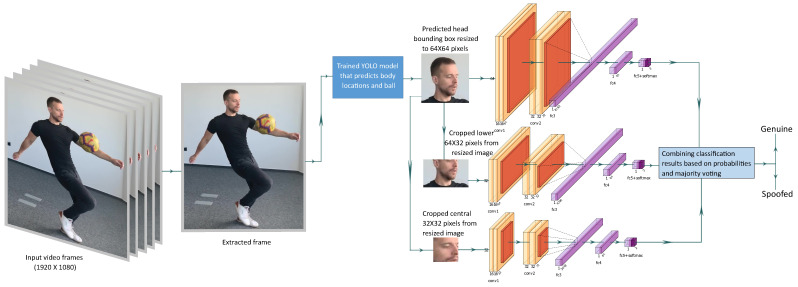
Our ensemble multi-stream model: frames are extracted from videos and one frame is processed at a time in three different streams. The outputs of the three streams are combined to derive the final classification results.

The ensemble model investigates the resized head bounding box in three different streams of processing over three spatial resolutions. The approach is presented in Figure 6. Similar to SF model, we consider one frame at a time. From the input HD video, single frames are extracted and fed to the trained YOLO model to extract the head region on the image. The detected head bounding box is resized to 64 × 64 × 3 pixels and processed in three different streams.

In the first stream, the resized head image is fed to the baseline model (same as SF). In the second stream, the lower 64 × 32 × 3 pixels of the resized head image are cropped and supplied to the modified baseline model for processing. On the initial convolutional layer of the baseline model, filters are modified to be of the size of the cropped image in this stream. This stream mostly obtains the visual data around the chin area and, in this way, bias coming from any users wearing eye glasses or caps is minimized. The last stream is fed with the cropped central 32 × 32 × 3 pixel data from the resized image. This data are processed by another modified baseline model that has the required initial convolution filter size. The stream processes the central crop of the face and this way it has no bias from any disturbing patterns from the background. The detections from these three streams are manually fused based on the detection probabilities and majority voting in order to arrive at an ensemble detection.

### 3.3. Learning

#### 3.3.1. Data Preparation and Pre-Processing

In order to learn the genuine and spoof cases, we used the same dataset used for training the YOLO body detection containing 50,500 Full HD images obtained from 38 users in 38 videos playing Sqiller. For training our models, we generated an additional 50,500 Full HD spoofed images using the original images by capturing the same videos on several monitors—a 27-inch Dell 4K monitor, a 15-inch Full HD Lenevo laptop monitor, and a 13-inch MacBook Pro monitor with the resolution of 2560 × 1600. Before training the networks, we pre-processed all video frames to extract the head region using the previously trained YOLO model and resize the head image to 64 × 64 pixels. We also used data augmentation techniques to reduce problems from overfitting—increasing and decreasing the pixel brightness by a value of 50, doubling and halving the image contrast, and adding Gaussian noise with variances of 50 and 100. These pre-processing steps were consistently added to all the video frames. Finally, before training, we shuffled the data and scale all the pixel intensities to the range [0, 1].

#### 3.3.2. Optimization

We used Adam [50] available in Keras to optimize our models with the initial learning rate of 1 × 10${}^{-4}$. The model was compiled with a binary cross entropy loss function. For training, we used a batch size of 12 samples and a 20% validation split. Models with improved validation accuracy were automatically saved every 100 epochs to retain best models.

## 4. Results and Discussion

### 4.1. Testing Scheme

**Figure 7:**
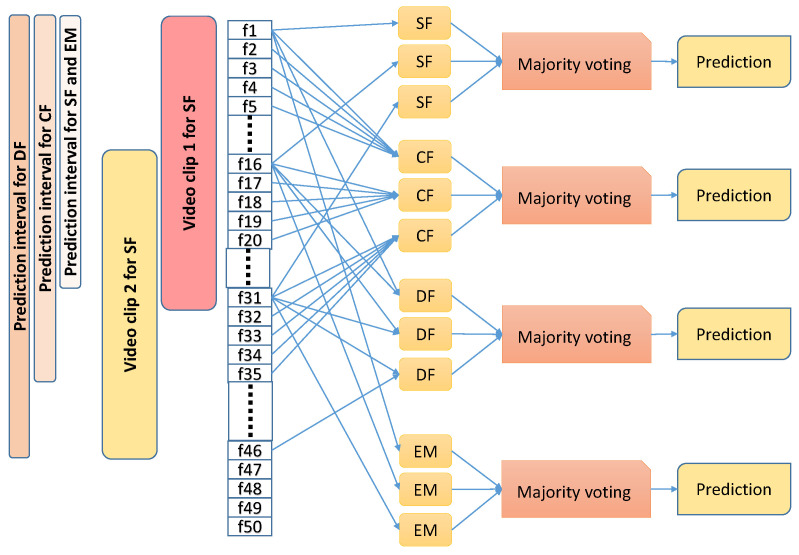
Schemes used for testing the proposed models: all models are tested on overlapping video clips. Within each video clip, three predictions are made and majority voting is applied to derive the prediction for this video clip.

Since we are aiming to detect spoof cases on a continuous basis either on recorded or on live video streams, we define chunks of overlapping video clips, each containing several frames (depending on the model under testing) with an overlap of 15 frames. For each clip, we provide a single prediction. To this end, within a considered clip, we run our models on three frames that are 15 frames apart and combine these predictions to obtain a single prediction for this clip. Therefore, given a video stream, in the case of SF and EM models, the first prediction is made after 31 frames and, thereafter, predictions are made every 15 frames. In the case of the DF model, since it takes as input two frames that are 15 frames apart, the first prediction is made only after 45 frames and, thereafter, predictions are made every 15 frames. In case of CF model, since it takes consecutive 5 frames as input, the first prediction is made after 35 frames and, thereafter, predictions are made every 15 frames. Figure 7 demonstrates the testing schemes more distinctly.

### 4.2. Experiments on Our Dataset

**Figure 8:**
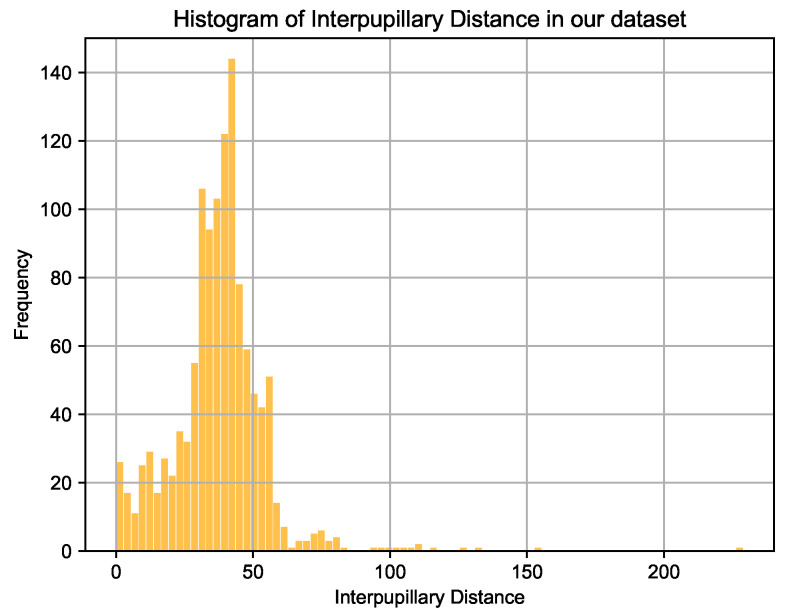
Histogram of interpupillary distances across various players in our database.

As mentioned earlier, to the best of our knowledge, our system is first of its kind exploring spoof detection from videos in the context of HAR. Since, also as mentioned before, we prepared a dataset containing 101,000 images, obtained from 38 different players. These images contain users located at varying distances from the camera so that all the body parts are visible. The face orientation of players widely varies across videos and sometimes the face is completely or partially occluded by the ball. We manually annotate these images with head bounding boxes containing facial and background information of the players which is then used for training a YOLO model that detects head regions in an image in real-time. Figure 8 shows a histogram of InterPupillary Distances (IPD) on all the images used for training our models (64 × 64). With the highest number of samples at around 44 pixels, our database also contains samples where IPD is evidently located between 0 and 50 pixels which shows how far away users are located from the camera. A value of 0 pixel IPD may mean either the face is not completely visible or a player is facing in an orthogonal direction to the camera.

#### 4.2.1. Training

We split the database by assigning 80% of the videos to the training set and 20% to the test set. To this end, we separated videos from eight players in our database and used them for testing. All the models were trained for 1000 iterations and training each model took about 24 h on average. Among the testing videos, predictions were made for each video clip containing 30 frames irrespective of the length of the video. To report results, we used results collectively from all video clips in the testing split.

#### 4.2.2. Video-Clip Level Predictions

We used videos from eight users (20% of the whole database) for testing that are not included in training. In these videos, users performed similar actions using a football as in the training set in challenging indoor and outdoor lighting conditions. The fake database for the test set was also created using random monitors that are mentioned in Section 3.3.1. Similar to the training set, the test set also contains videos from users who are both male and female in the age group 8–40. We report our results using a set of metrics commonly considered in bio-metric presentation attack evaluation—Attack Presentation Classification Error Rate (APCER), Bona fide Presentation Classification Error Rate (BPCER) and Half-Total Error Rate (HTER) [51]. APCER and BPCER are analogous to False Acceptance Rate (FAR) and False Rejection Rate (FRR). Thus, lower values of APCER, BPCER and consequently, HTER, indicate better performance of a model. Table 2 presents results of the various described models on the testing split. To report these results, the outputs of the models were used as obtained and thresholding based on class prediction probabilities was not applied.

**Figure 9:**
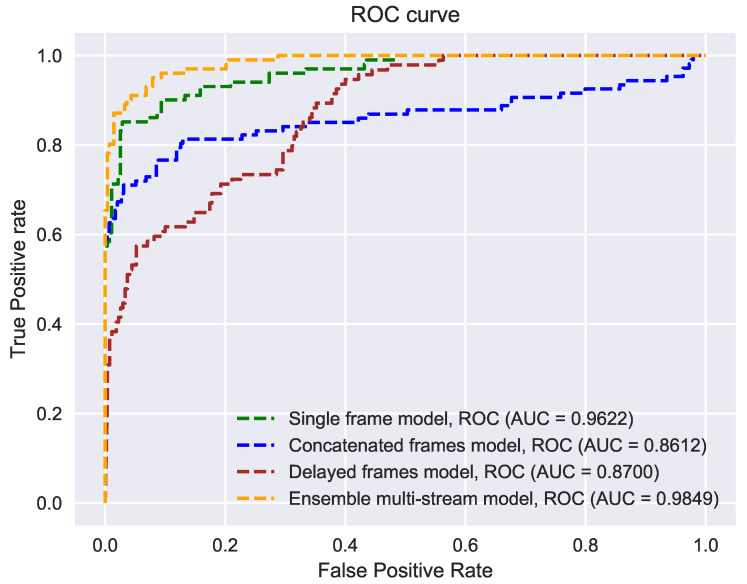
Receiver operating characteristics (ROC) of the methods evaluated on our testing database. Compared to other models; the ensemble multi-stream model produced better classification results.

The ensemble model outperformed other methods by a considerable margin. This can also be validated by analyzing the trade-off between False Positive Rate (FPR) and True Positive Rate (TPR) on the testing set (see Figure 9). (a Higher value of the Area Under Curve (AUC) represents better performance of a model). Note that the single frame method also provides reliable results. However, our experiments show that combining the frames across time did not yield better results. Particularly, in the case of the CF model, the APCER value is high, indicating that several fraud users are accepted as valid users by this model. This suggests that combined frames may not always contain patterns that can be sufficiently characterized by our deep learning model and may be completely arbitrary. In the case of the DF model, the BPCER value is high, indicating that several real users were categorized as fake users by this model. From this behavior, we hypothesize that the DF model learned features relating more to motion than to the patterns related to spoofing detection. It is important to note that our detected head region contains visual information about the background or the scene as well as the user costume. The cropped lower region contains less visual information about the background, but contains information about the user costume and thhe cropped central region contains mainly visual information of the user face. We observed that specular, glossy surfaces in the background, spectacles and face masks of users, and dense striped costumes can mislead spoofing detection. By utilizing three cropped regions extracted from the detected head bounding box, we believe that the EM makes smaller errors in spoofing detection than other methods used for comparison.

#### 4.2.3. Comparison with Existing Baseline Spoofing Detection Methods

To assess the effectiveness of our ensemble method, we considered recent baseline spoofing detection methods for comparison. Please note that, as pointed out earlier, there are no relevant data-driven approaches in the literature for spoofing detection from videos when users are performing simple or complex activities and are free to choose their position. Particularly, we consider the following four descriptors for the experiment, which are reported as potential candidates in the literature for spoofing detection applications using facial image data: LBP histograms, Local Binary Pattern histograms from Three Orthogonal Planes (LBP-TOP) [52], Statistical Binary Pattern histograms (SBP) [53] and Statistical Binary Pattern histograms from Three Orthogonal Planes (SBP-TOP) [54]. Moreover, it is reported in the literature that Support Vector Machine (SVM) classifiers achieved better accuracy than random forest classifiers in spoofing detection applications [54].

Consequently, for comparison, we trained four different SVMs using the abovementioned four descriptors. For training SVMs, We used the scikit-learn toolkit. While calculating these descriptors, we use radius, R = 1 and number of points P = 8 (see [54] for more details on these parameters). To calculate SBP, we considered two orders of moments obtained with the mean and variance. To compute the orthogonal planes, we considered 3D frame stacks containing 20 consecutive video frames. For acquiring joint histograms in the case of SBP and SBP-TOP, as described in [54], two binary images are computed additionally by thresholding the input or moment images with respect to their average value. For testing, we used the same testing dataset that is used for comparing the proposed methods. Table 3 presents the results from the baseline methods in the literature together with the results from the EM method using our testing dataset. Experimental results show that our EM method outperformed other methods by a considerable margin. Our observations correspond to the results presented in [36]—that approaches using low- or medium-level texture descriptors and training simple classifiers using these descriptors for spoofing detection are not effective under cross-dataset protocols.

#### 4.2.4. Experiments with Video Compression

As mentioned earlier, depending on the complexity of the action to be recognized, it is necessary to stream the video to a remote server where actual processing of the video stream happens. In cases like motion-driven virtual games, where user videos are streamed from a mobile telephone to a remote server, there is not always enough network bandwidth to stream the video in its native quality or resolution. Subsequently, video compression techniques are applied to reduce the video bitrate which introduces video artifacts. To evaluate the ability of our EM model to correctly classify the spoof cases, we generated compressed video streams with varying bitrates from 300 kbps to 1500 kbps.

**Figure 10:**
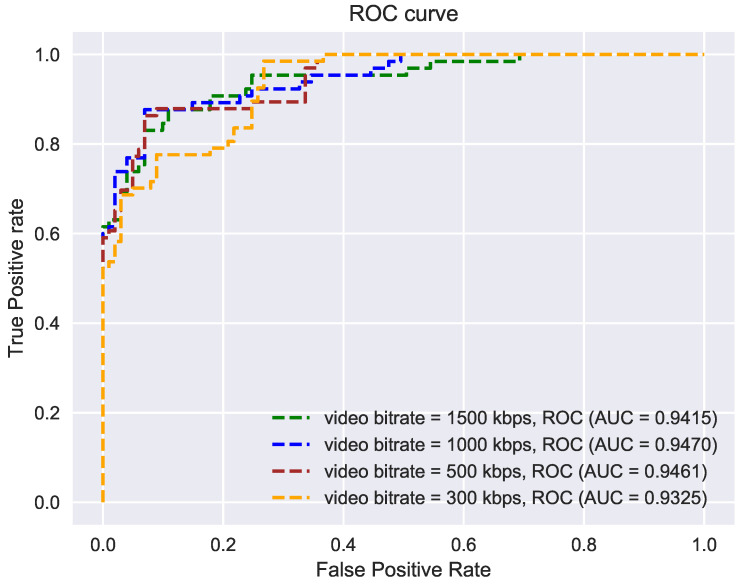
Evaluation of sensitivity of our ensemble multi-stream model to video compression rates: the model produces robust results even at lower bitrates (higher compression).

For this experiment, a recorded video was considered and evaluation was performed completely offline. Multiple videos were generated with different bitrates using ffmpeg [55]. Figure 10 shows the AUC values under various bitrates. Results show that our ensemble model performs robustly even under extreme compression (300 kbps).

### 4.3. Experiments with Face Recognition Databases

To evaluate the ability of our spoofing detection model to generalize to other domains such as face recognition systems, we experimented with two widely known datasets in this context: Idiap REPLAY-MOBILE [56] and CASIA Face AntiSpoofing [37]. In particular, we identified the spoofing cases that occur in the video replay cases and evaluated the performance of our algorithm on these attacks. The CASIA Face AntiSpoofing Database consists of 600 video clips of 50 subjects. Out of the 600 video clips, 150 clips represent video replay attacks. Compared to the Idiap database, the CASIA DB provides images captured using a variety of cameras (Sony NEX-5-HD, two low quality USB) to capture replay attacks displayed on an iPad. However, a significant deficiency of this database is that the video replay attacks are captured in very low resolution (640 × 480). The REPLAY-MOBILE dataset consists of 1190 video clips of photo and video presentation attacks (spoofing attacks) to 40 clients, under different lighting conditions. These videos were recorded with an iPad Mini2 (running iOS) and an LG-G4 smartphone (running Android) in full HD resolution.

Table 4 shows the evaluation results on testing sets of the considered face recognition databases. The facial images were cropped using the supplementary data obtained from the database and then resized to 64 × 64 pixels before being fed to our EM model. The test results are reported using our testing scheme (see Figure 7). Our results show that our approach performs well on the REPLAY-MOBILE database irrespective of the fact that the users are located very close to the camera (higher IPD values than that of our database). Our method also performs reasonably well on the CASIA database. The relative drop is the performance in case of the CASIA database is due to the low resolution input images. Note that our method is trained on image data that are extracted from full HD input images and we hypothesize that our learned feature set does not correspond very well to images that are captured at lower resolution. Note that this may not be a problem since the current generation sensors used in HAR applications, including surveillance systems, are capable of capturing full HD images.

### 4.4. Mobile Implementation and Performance

We implemented our Ensemble multi-stream model in swift for IOS [57]. The application is standalone and tested on iPhone 8 released in 2017 and has a 2.39 GHz hexa-core 64-bit. Our Keras models as well as the YOLO model for head bounding box detection are converted to CoreML API for running on the IOS device. The application takes as input incoming frames from the on-device-camera and runs our trained YOLO model to detect head bounding box information. The head bounding box is then resized to 64 × 64 for ensemble multi-stream processing. We follow the strategy shown in Figure 7 for processing the incoming frames for detection. The algorithm works in real-time.

The proposed method can run on an iPhone 8 and processes a single frame on an average in 4 ms. This translates to a frame rate of about 250 frames per second. Following our testing scheme shown in Figure 7, running the EM every 15 frame further ensures that there are enough resources left on the device to run the activity recognition applications.

### 4.5. Performance Constraints

We compute head bounding box information using a pre-trained YOLO model with fixed anchor sizes for bounding boxes. The anchor sizes are chosen assuming that a user is located at a distance range 0.5–2.5 m away from the camera. In addition, for training we use those samples when a user is located within this range. If a user moves out of this range, bounding boxes may still be detected, but with less probability. However, due to fixed size anchors, in such cases the bounding box would contain relatively more information about the background. Our algorithm fails to detect spoofing in such a situation.

Our spoofed database contains samples captured from cameras held at angles close to zero (almost held vertically) along the camera’s Z axis (depth axis). Our rigorous testing shows that if the recording camera (the camera used for action recognition and not the original camera that the captured video for spoofing) rotates beyond the range −15 to 15 degrees along the camera’s Z axis, our algorithm fails to detect spoofing attacks.

Moreover, our database consists of images of a user where at least one of the eyes is visible (the user can face sideways perpendicular to the direction pointed by the camera) or the face is covered with a football which is our considered object for interaction. If it happens that a user faces completely away from the camera (only hair or a bald head is visible), our algorithm also fails to detect spoofing in such cases.

Under natural lighting conditions, our EM method can precisely detect spoofing cases. To deal with artificial lighting conditions, we also captured enough samples in our database, where a scene is lit by a bulb with a flickering frequency observable by cameras. However, thorough testing shows that if more than one light source is in the scene, our algorithm has issues detecting the spoofing cases.

## 5. Conclusions and Future Work

In this work, we studied video replay spoofing detection in applications where human activity recognition using RGB sensors plays a crucial role. Outdoor weather and visibility conditions are not controllable and therefore spoofing detection under such circumstances is a difficult task. We believe that, to the best of our knowledge, we are studying this problem for the very first time in HAR domain. In particular we considered the virtual football game—SQILLER application to experiment with various designed models. We formulated an ensemble multi-stream model that detects spoofing cases arising from video replay attacks. Our model inspects distinct regions of human faces and combines these observations to provide robust classification between spoof and genuine cases. We collected a database comprising of 38 subjects in widely varying lighting conditions and backgrounds. We also generated corresponding spoof videos using several monitors to train and test our models.

We showed that our model provides robust results even if the face of the subject is partially visible. We also evaluated the performance of our trained model on compressed videos at different bitrates and the ability to generalize to face recognition systems. However, face recognition may need fine tuning of our model to better fit to such cases where IPD at full HD resolution is higher than that of our training images.

We also implemented and validated our algorithm on a mobile device and showed that our approach can work in real-time with minimal memory footprint, leaving enough room to run the activity recognition algorithm alongside. In future, our method can be easily adapted to mobile devices containing advanced sensors like depth sensors, and this would further improve the performance of our spoof detection approach. In addition to replay attacks, in future we also intend to study and explore algorithms for detecting masks or hoodies covering the face and/or other clothing that cover body parts and are designed for confusing spoof detection systems.

## Figures and Tables

**Table 1:** Performance of published methods on face spoofing detection. Please refer to the text concerning the metrics used for evaluation.

Method	State-of-the-Art Performance
Face motion analysis	**ZJU Eyeblink** [13] (Intra-DB, Cross-DB) [13]: (95.7, n/a)
	**Idiap Replay-attack** [19] (Intra-DB, Cross-DB) [12]: (1.25, n/a)
	**CASIA FASD** [37] (Intra-DB, Cross-DB) [38]: (21.75, n/a)
Depth analysis	**Idiap Replay-attack** (Intra-DB, Cross-DB) [39]: (12.5, n/a)
Image texture analysis	**Idiap Replay-attack** (Intra-DB, Cross-DB) [36]: (15.54, 47.1); [40]: (0.8, n/a); [41]:(2.9, 16.7); [42]:(1, n/a)
	**CASIA FASD** (Intra-DB, Cross-DB) [41]: (6.2,37.6); [42]: (7.2, 30.2)
Image quality analysis	**Idiap Replay-attack** (Intra-DB, Cross-DB) [43]: (7.41, 26.9); [25]: (15.2, n/a);
	**CASIA FASD** (Intra-DB, Cross-DB) [43]: (12.9, 43.7)
	**MSU MFSD** [43] (Intra-DB, Cross-DB) [43]: (5.82, 22.6)
Frequency domain analysis	**Idiap Replay-attack** (Intra-DB, Cross-DB) [32]: (2.8, 34.4)
	**CASIA FASD** (Intra-DB, Cross-DB) [32]: (14, 38.5)
	**UVAD** [32] (Intra-DB, Cross-DB) [32]: (29.9, 40.1)
Deep learning based methods	**PR FASD** [44] (Intra-DB, Cross-DB) [44]-ResNet: (n/a, 14.19); [44]-DenseNet: (n/a, 16.97)
	**MSU MFSD** (Intra-DB, Cross-DB) [44]-ResNet: (n/a, 20.53); [44]-DenseNet: (n/a, 21.78)
	**Idiap Replay-attack** [19] (Intra-DB, Cross-DB) [44]-ResNet: (n/a, 31.42); [44]-DenseNet: (n/a, 31.52)

**Table 2:** Results (%) on our testing database.

Method	APCER	BPCER	HTER
SF	9.9010	12.9496	11.4253
CF	36.4486	1.7007	19.0746
DF	3.1915	45.5556	24.3735
EM	8.9109	6.1151	**7.5130**

**Table 3:** Results of existing baseline spoofing detection methods (%) on our testing database after training using our training database.

Method	APCER	BPCER	HTER
LBP	40.5780	48.1586	44.3683
LBP-TOP	27.2727	40.3226	33.7977
SBP	**3.1915**	45.5556	24.3735
SBP-TOP	15.4124	78.9660	47.1892
EM	8.9109	**6.1151**	**7.5130**

**Table 4:** Results (%) on considered face recognition databases.

Method	APCER	BPCER	HTER
REPLAY-MOBILE	5.4678	13.7856	9.6267
CASIA	27.0916	16.0396	21.5656

## Data Availability

The data presented in this study are available on request from the corresponding authors. The data are not publicly available due to licensing and GDPR authorisation reasons.
